# Molecular mechanisms associated with effects of hydrogen molecule in liver diseases: the review of current evidence

**DOI:** 10.1186/s40001-025-03347-z

**Published:** 2025-11-05

**Authors:** Qian Zhu, Xianzhi Li, Shiyang Li, Shunjin Liu, Yunyun Tian, Xiangyi Xing, Li Yin

**Affiliations:** 1 Clinical Medical Research Center, Meteorological Medical Research Center, Panzhihua Central Hospital, No. 34 Yikang Street, East Panzhihua City, Sichuan, 617000 China; 2 Department of Geriatric Medicine, Panzhihua Central Hospital, Sichuan, 617000 China; 3 Key Laboratory of Precision Medicine and Clinical Transformation of Geriatric Diseases, Panzhihua Central Hospital, Sichuan, 617000 China

**Keywords:** Hydrogen molecule, Oxidative stress, Inflammation, Glycolipid metabolism, Cytoprotection

## Abstract

Oxidative stress and inflammation play a key role in the occurrence and progression of liver diseases, inducing hepatocyte apoptosis, fibrosis, and even cancer. However, there is a lack of effective therapeutic interventions to slow liver inflammation and the progression of metabolic liver diseases. The unique biological properties of molecular hydrogen’s selective scavenging of pathological free radicals have shown therapeutic potential in animal studies and clinical trials of chronic liver diseases. This review describes innovative technologies and applications of molecular hydrogen in the treatment of liver injury and liver metabolic diseases, focusing on its mechanisms of action in regulating redox and inflammatory cascade signal transduction and specifically discussing its potential for glucolipid metabolic homeostasis and intestinal microbiota remodeling, as well as cell protection. Future studies are needed to further identify the unknown mechanisms by which molecular hydrogen improves the beneficial role of the liver microenvironment and advance the clinical application of hydrogen therapy.

**Supplementary Information:**

The online version contains supplementary material available at 10.1186/s40001-025-03347-z.

## Background

The updated “Global Burden of Liver Disease Report in 2023” shows that liver disease causes about 2 million deaths worldwide each year, accounting for 4% of total deaths [1]. The Asia–Pacific region, in particular, has a large population and bears most of the global burden of chronic liver disease-related diseases. Nearly 50% of the world's chronic liver disease, cirrhosis, and liver cancer-related deaths occurred in the Asia–Pacific region [2]. According to the "Global Hepatitis Report 2024" recently released by the World Health Organization, the number of deaths due to viral hepatitis is increasing globally, with 1.3 million deaths per year, and has become the second largest infectious cause of death in the world [3, 4]. Maintaining liver health is an important way to reduce disease burden and prolong life.

In recent years, many laboratory and clinical studies have shown the promise of hydrogen application in the treatment of a range of acute and chronic liver diseases. Hydrogen molecule has high biofilm penetration and intracellular dispersion ability, and can enter various organelle structures in the nucleus and cytoplasm. In 2007, Ohsawa et al. [5] reported the landmark finding that hydrogen (> 25 μM) could reduce the levels of peroxynitrite anion and hydroxyl radical in ischemic stroke model. The solubility of hydrogen in the liver was as high as 43 μM in the microsensor study of living adult mice [6]; and the liver had the highest H_2_ concentration after supplementing exogenous H_2_ by various strategies [7]. It shows that the retention concentration of hydrogen in the liver is sufficient to exert its antioxidant effect and provide the basis for elucidating the potential mechanism of hydrogen’s beneficial effect on the liver. In this review, we comprehensively summarize the overview of reports regarding the potential role of molecular hydrogen therapy in liver diseases, including hepatic steatosis, non-alcoholic steatohepatitis (NASH) or metabolic dysfunction-associated liver disease (MASLD), viral hepatitis, liver ischemia–reperfusion injury (IRI), and liver damage or dysfunction due to cancer chemoradiotherapy or other causes.

## Technological innovation of hydrogen molecule’s delivery

Under physiological conditions, the human body's endogenous H_2_ mainly comes from the gut microbiota. H_2_ metabolism homeostasis of intestinal flora can affect liver disease progression. Studies have demonstrated that endogenous H_2_ produced by intestinal flora can inhibit concanavalin A-induced hepatitis, while antibiotic inhibition of intestinal flora can aggravate hepatitis injury [8]. Exogenous intake of hydrogen-producing substrates, such as 20% high amylose cornstarch [9], lactulose [10], and L-arabinose [11], significantly improved acute liver IRI, hepatic steatosis, and accelerated liver regeneration in animals. The common ways of exogenous hydrogen delivery include hydrogen inhalation, drinking hydrogen-rich water (HRW), and injecting hydrogen-rich saline (HRS).

It is worth noting that hydrogen molecules have low water solubility and high but aimless diffusivity, and it is difficult to predict the effective concentration of hydrogen at a specific location and the possible impact on other organs. Targeted transport, controlled release, and long-term accumulation of hydrogen are common challenges in hydrogen medical research. To address this problem, some scientists have recently designed vectors that can steadily release H_2_ at local targets. For example, coral calcium hydride (CCH) is a porous powder made of coral calcium reacting with hydrogen at high temperatures of 800–950℃ for about 2–12 h. Chen et al. [12] and Ma et al. [13] used CCH as a hydrogen-rich carrier and confirmed that the hydrogen produced by CCH could improve NASH and MASLD. However, as a dietary supplement, the experimental evidence on human toxicity of CCH is limited. Its strongly alkaline hydrolysates may cause serious tissue damage, and the toxicity needs to be carefully evaluated. Nanomaterials showing excellent biosafety offer prospects for targeted high-dose delivery of molecular hydrogen. Wu et al. [14] constructed an X-ray triggered sustainable in situ H_2_ producing platform Au-TiO2@ZnS, which achieved excellent therapeutic effects and low inflammatory side effects in combination with radiotherapy for in situ liver cancer in mice. He et al. [15] developed the N-(3-triethoxysilylpropyl) gluconamide (Glu)-modified magnesium silicide (Mg2Si) nanosheets (MSN-Glu), which can target hepatocytes by actively recognizing the asial glycoprotein receptor on hepatocytes. The developed MSN-Glu nanomedicine exhibited a high hydrogen delivery capacity of 105 mg/g, which is 6.6 × 10^4^-fold higher than that of saturated HRW, and its time duration of sustained H_2_ release was as long as eight days under physiological conditions in great favor of high-dose H_2_ delivery. They have also developed a Pd nanoparticle, which accumulates in the liver in a targeted manner post-intravenous injection, capable of rapidly capturing H_2_ passing through the liver, storing it in a solid form of Pd hydroxide (PdH), and finally catalyzing ·OH for local and high-efficacy anti-chronic liver diseases [16]. The development of materials science and nanotechnology, combined with targeted drug delivery technology, is also the driving force for future hydrogen medical technology innovation. However, there are still many difficulties and challenges in obtaining a stable biological response and precise treatment of nano-hydrogen drugs. For example, the introduction of foreign nanoparticles may increase the complexity of drug delivery and release diffusion, while the biological effects of nanocarriers on cells, tissues, and organs during transport in vivo are unknown, which poses many obstacles to further clinical research.

## Animal experiments and clinical applications of hydrogen molecule

The accumulating literature substantiates the potential of hydrogen to regulate liver homeostasis, and the molecular effects of hydrogen under physiological and pathological conditions have been noted. In healthy conditions, long-term hydrogen intervention causes extensive changes in physiological function throughout the body. Body weight and serum biochemical indexes, including blood lipid, blood glucose, liver enzymes, and sterol lipids, changed significantly at different time points [17]. Within the liver tissue, inflammation-related pathways and metabolic remodeling of lipid and amino acid breakdown are triggered, of which the NADP/NADPH redox pathways may be the central regulator [18, 19]. HRW intake may also promote the flow of toxic substances into the bile by enhancing* p*-glycoprotein and Mrp2 protein expression [20]. According to the current research results, H_2_ does not cause health threats or liver damage to the body under the condition of health, but for the "reasonable application" of hydrogen, it is necessary to consider the duration, concentration, and interaction with specific target organs, and there is still a large gap to be filled in the applicability of hydrogen to healthy people.

The application of molecular hydrogen therapy has been studied in animal models of various liver diseases, and the relevant evidence and mechanisms are fully summarized in Table SI. Based on these reported experimental data, the effects of hydrogen appear to be beneficial in the models of liver steatosis, NASH/MASLD, liver IRI, or liver injury due to other causes, with histopathological damage of the liver being alleviated, and its recognized antioxidant and anti-inflammatory properties playing a significant role.

The reports on the application of molecular hydrogen therapy in clinical liver disease are summarized in Table SII. In clinical patients with MASLD, drinking HRW or inhaling a hydrogen and oxygen mixture can improve serum liver enzymes, reduce liver fat accumulation and degeneration, and improve oxidative stress and inflammation indicators [21, 22]. But other studies have also reported that these indicators seem to show only a favorable trend, and the absolute change is not significant [23, 24]. There is not enough evidence to tell whether it is fake progressive remission or whether hydrogen actually works. Adjuvant therapy with HRW can improve the platelet mitochondrial bioenergy function of MASLD patients and increase the concentration of CoQ10 in platelets [24]. HRW could be a promising strategy for mitochondrial health recovery in patients with MASLD. In addition, HRW significantly reduced viral DNA levels in patients with hepatitis B [25], and improved liver function and quality of life in patients undergoing cancer radiotherapy and chemotherapy [26, 27]. A growing body of data seems to confirm the evidence of the conceptual benefit of hydrogen in the treatment of liver disease. As the basic research of hydrogen medicine tends to saturate the types of diseases, and the need for the transformation and application of hydrogen medicine, high-quality clinical research is gradually receiving attention. However, little high-level clinical trial evidence has been published on the use of molecular hydrogen to treat liver disease, and some studies are still being registered. The role of molecular hydrogen in liver disease prevention and long-term disease remission still needs to be formally demonstrated in rigorously designed, high-quality clinical trials and actual practice.

Future studies should also clarify whether hydrogen therapy is effective in specific liver disease groups, such as patients with liver cancer or viral hepatitis after targeted therapy or chemoradiotherapy. In addition, the optimal timing of the application of hydrogen therapy remains to be determined, and the maximum benefit of hydrogen therapy in a preventive setting, during or after treatment or as a neoadjuvant or combination therapy is highly awaited. In the past, whether it is basic research or clinical research, hydrogen medicine has paid more attention to the treatment of liver disease. The use of hydrogen may ideally be integrated into the whole life cycle in the form of daily lifestyle, and the direction of hydrogen medical research needs to be tilted towards disease prevention. Another unmet need is the identification of molecular hydrogen prognostic markers, which may justify early treatment with hydrogen therapy in certain patient populations to prevent complications, and targeted response markers, since it is currently impossible to predict whether a patient will respond to a given treatment.

## Underlying mechanisms of hydrogen molecule’s protective effect

**Fig. 1:**
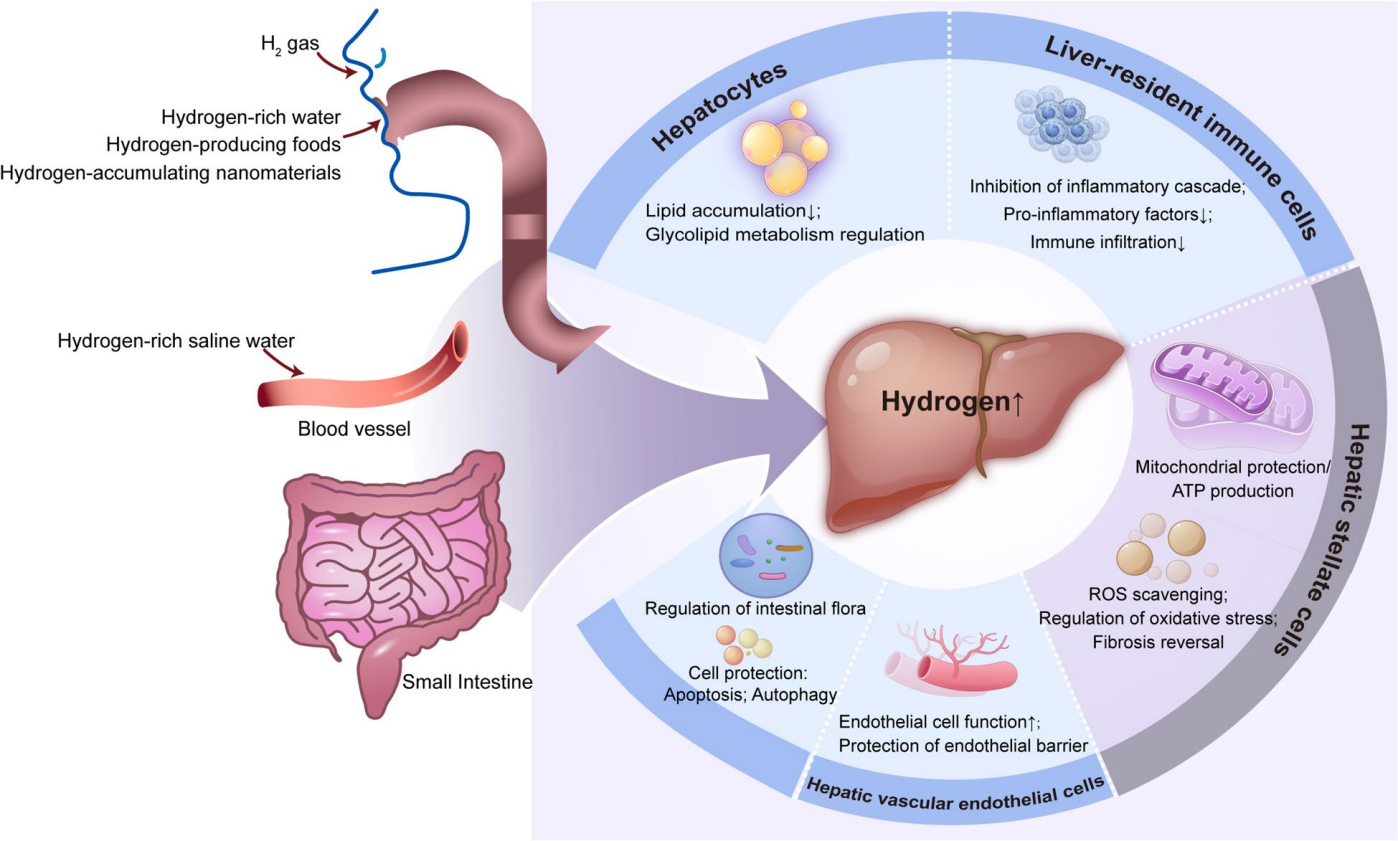
Potential mechanisms associated with molecular hydrogen alleviating progression of liver diseases. ROS reactive oxygen species; ATP adenosine triphosphate

Liver injury is a class of diseases with a progressive decline in liver function caused by a variety of pathogenic factors, such as drug-induced, toxic, traumatic, ischemic, or infectious injury. In Fig. 1, we summarize the potential mechanism of molecular hydrogen in the application of liver diseases. The core of its pathophysiology involves the clearance of free radicals, the inhibition of inflammatory cascade reactions, the remodeling of glycolipid metabolism, cell death, and the regulation of the intestinal microbiota.

### Cellular targeting of molecular hydrogen's actions

In liver injury models, the actions of H₂ involve multiple key cell types in liver tissue, and its protective effects are achieved by regulating the functional status of these cells. Hepatocytes are the primary functional cells of the liver, responsible for core physiological functions such as metabolism and detoxification, and are also the most susceptible cell type to liver injury. Molecular hydrogen directly protects hepatocytes by scavenging excessive reactive oxygen species (ROS) within them and reducing lipid accumulation [28]. Liver-resident macrophages (Kupffer cells) are central players in innate immunity [29]. Molecular hydrogen can inhibit the excessive activation of Kupffer cells, specifically by reducing the secretion of pro-inflammatory factors (e.g., tumor necrosis factor-α (TNF-α), interleukin(IL)-1β) to mitigate local inflammatory responses; and downregulating inflammatory chemokines to suppress the migration of neutrophils to liver injury sites, thereby alleviating inflammation-mediated tissue damage. Hepatic stellate cells are key effector cells in hepatic fibrosis. Persistent liver injury triggers their activation (differentiation into myofibroblasts), which synthesize large amounts of extracellular matrix (e.g., collagen), leading to hepatic fibrosis and even cirrhosis. In NASH models, molecular hydrogen reverses hepatic fibrosis by inhibiting the phosphorylation of signal transducer and activator of transcription 3 (STAT3), the activation of downstream mitogen-activated protein kinase (MAPK) signaling, and regulating oxidative stress–inflammatory pathways (e.g., nuclear factor-κB [NF-κB]), thereby reducing hepatic stellate cell activation and collagen deposition [30]. Hepatic vascular endothelial cells form the hepatic sinusoidal barrier. In models such as IRI and acute cholangitis, endothelial cell damage increases vascular permeability and causes microcirculatory dysfunction, exacerbating liver ischemia and inflammation. Molecular hydrogen protects endothelial cell function by inhibiting NF-κB activation and reduces endothelial barrier disruption by suppressing the activity of matrix metalloproteinases (MMP2/9) and regulating tight junction proteins, among other mechanisms. In summary, the actions of molecular hydrogen in liver injury models exhibit cellular targeting, and this multi-cellular regulatory property constitutes the core mechanism underlying its protective effects across various liver injury models.

### Free radical-related redox and oxidative stress

When the body’s redox homeostasis is out of balance, the oxidative capacity is greater than the antioxidant capacity, and the imbalance between the production and removal of ROS and reactive nitrogen species (RNS) in the body leads to oxidative stress, which in turn leads to liver cell damage, inflammation, and liver fibrosis. In animal models of acute and chronic liver injury, hydrogen alleviates excessive accumulation of ROS and significantly reduces markers of oxidative damage, such as DNA oxidative damage end product 8-hydroxy-2-deoxyguanosine (8-OHdG) [31], protein oxidative damage end product 3-Nitrotyrosine(3-NT) [32], lipid peroxidation end product malondialdehyde (MDA) [33] and 4-hydroxy-nonenal (4-HNE) [34], which significantly improved liver enzyme indexes and morphological liver injury, improved indicators of liver fibrosis, such as Acta2/α-SMA [16] and TGFβ/SMAD [30], and promoted liver regeneration after transplantation. In terms of mechanism, molecular hydrogen activates the activities of key antioxidant enzymes, such as superoxide dismutase (SOD), catalase (CAT), and glutathione peroxidase (GPx), which promote ROS clearance [35]. Cell signaling and gene expression are finely regulated by redox regulation, and many pathways of signal transduction respond to changes in cell redox balance. Molecular hydrogen inhibits the formation of ROS or repairs the ROS-caused damage by regulating redox-sensitive molecules and signaling pathways. For example, molecular hydrogen activates nuclear factor 2-related factor 2 (NRF2) and its downstream antioxidant enzymes SOD1/2 and anti-oxidative stress protein heme oxygenase 1 (HO-1) to alleviate hypoxia reoxygenation injury and hepatic steatosis in hepatobiliary duct cells [36, 37]. NF-κB, MAPK, and c-Jun N-amino-terminal kinase (JNK) signaling pathways are also sensor–effector mechanisms of oxidative stress signaling. The effect of hydrogen on the activity of these molecules has been observed in liver disease [30, 36, 38].

Mitochondrial respiratory chain is the main source of ROS production, and high local concentration of ROS can lead to DNA mutation directly disrupting mitochondrial dynamics. Molecular hydrogen has been found to be associated with mitochondrial respiratory function and ATP production in the liver of mice with obstructive jaundice [39]. CCH has been observed to reverse liver RCR abnormalities and proton leakage, restoring the function of mitochondrial complexes II and IV [12]. The primary function of NAD(P)H quinone dehydrogenase 1 (NQO1) in the cell is to convert NADH and NADPH into NAD+ and NADP+, protecting cells from oxidative stress by converting quinone to hydroquinone. Previous studies have shown that the application of molecular hydrogen is related to the upregulation of NQO1 in the liver, and NQO1 is also a detoxification enzyme regulated by NRF2 [33]. Iron porphyrins are also the molecular hydrogen-targeting molecules and redox-related biosensors that work with H_2_ reactions to inhibit free heme-catalyzed Fenton-like reactions to prevent non-alcoholic fatty liver disease [15]. The potential of hydrogen to restore redox balance in the liver, reduce oxidative stress, and possibly slow the progression of liver injury highlights the stabilizing effect of hydrogen on free radicals. ROS and RNS are the core roles of redox and are collectively referred to as endogenous free radicals. In the narrowest sense, there are four types of ROS: single oxygen(^1^O_2_), superoxide anion(O_2_^·−^), H_2_O_2_ and hydroxyl radicals (HO) [40]. RNS refers to free radicals and nitro compounds with high oxidation activity generated by the interaction of NO with targets inside and outside cells, including ONOO· and its protonic form peroxynitrite HOONO [41]. In terms of inducing apoptosis, RNS is superior to most ROS due to its peroxidation and nitrification. In 2007, Oshawa et al. [5] found that H_2_ can selectively neutralize the most cytotoxic reactive oxygen species (HO· and ONOO·) in the brain IRI model without affecting other physiological reactive oxygen species. As far as we know, most studies in clinical and animal liver injury models at present focus on the overall clearance effect of molecular hydrogen on ROS, and only He et al. clearly demonstrated the HO· clearance effect of molecular hydrogen on mouse liver AML-12 cells [15, 16]. Some previous studies have found significant differences in ROS/RNS levels and their mediated oxidative stress levels across liver injuries [42–44]. It remains to be seen which free radical dominates in different liver injury models, whether hydrogen removal of HO· fully explains the antioxidant effects of hydrogen, and whether hydrogen also has selective removal effects on other reactive free radicals.

### Inflammatory signal transduction and response

**Fig. 2:**
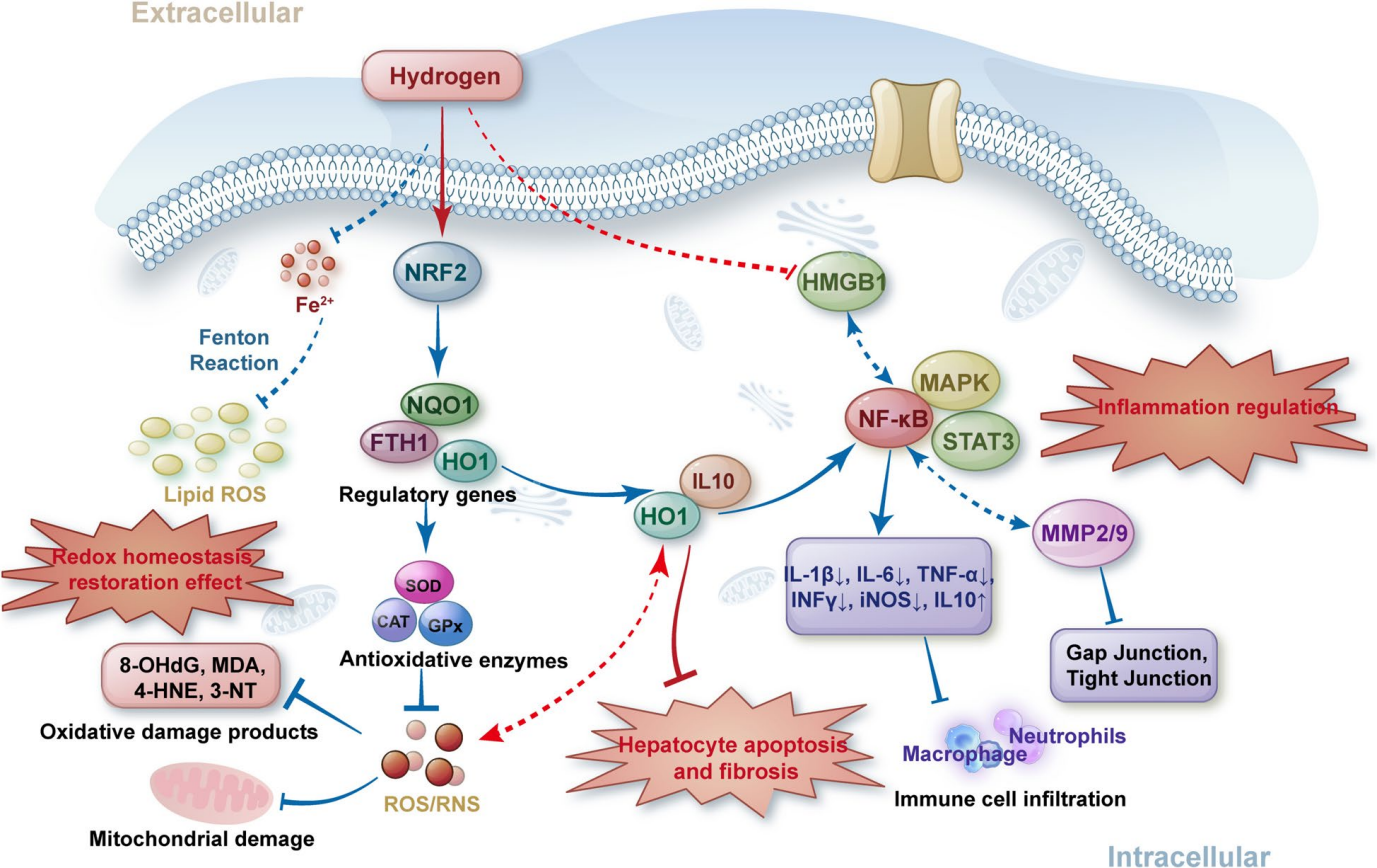
The interactive regulatory mechanism of molecular hydrogen on liver oxidative stress and inflammatory cascade reaction. CAT catalase; FTH1 ferritin heavy chain 1; GPx glutathione peroxidase; HMGB1 high mobility group box 1 protein; HO1 heme oxygenase 1; IL interleukin; iNOS inducible NOS; INFγ interferon-γ; MAPK mitogen-activated protein kinase; MDA malondialdehyde; MMP2/9 matrix metallopeptidase 2/9; NF-κB nuclear factor-κB; NRF2 NUCLEAR factor 2-related factor 2; NQO1 NAD(P)H quinone dehydrogenase 1; ROS reactive oxygen species; SOD superoxide dismutase; STAT3 signal transducer and activator of transcription 3; TNFα tumor necrosis factor α; 3-NT 3-nitrotyrosine; 4-HNE 4-hydroxy-nonenal; 8-OHdG 8-hydroxy-2-deoxyguanosine

Anti-inflammatory effects are another recognized mechanism of molecular hydrogen. Molecular hydrogen exerts anti-inflammatory effects by regulating cytokines and inflammatory mediators in circulation and tissue, such as IL-1β, IL-6, IL-10, TNF-α, inducible NOS (iNOS), interferon-γ (INFγ), and vascular endothelial growth factor (VEGF), reducing the infiltration of macrophages and neutrophils in liver lesion tissues [8, 13, 35].After the body's inflammatory response is triggered, sensors on the surface of immune cells recognize and bind to these inflammatory inducers, inducing cascade transduction of a variety of classical inflammatory signaling pathways, including NF-κB, MAPK, STAT3, etc. Molecular hydrogen interacted with the cross talk of the HO-1/IL-10 axis and suppressed the expression of phosphorylated STAT3 and the activation of downstream pMAPK signaling to reverse hepatocyte apoptosis as well as hepatic inflammation and fibrosis in NASH specimens [30]. The activation of NF-κB was significantly inhibited in HRS-treated animals after a partial hepatectomy or liver ischemia/reperfusion [45, 46]. Molecular hydrogen is involved in the regulation of these classical inflammatory signaling pathways in liver diseases, and these transcription factors are also regulatory targets of oxidative stress. Inflammatory and oxidative stress are mutually induced and promoted. Reactive oxygen intermediates are thought to be a second messenger involved in NF-κB activation of TNFα and IL-1β [47]. Activation of the NF-κB signaling pathway may establish the correlation between inflammation and oxidative stress in liver disease, but whether NF-κB is the central pathway for molecular hydrogen regulation of inflammation and oxidative stress in the liver and synergies with other inflammatory signals is unclear (Fig. 2). High mobility group box 1 protein (HMGB1) is a signal transmitter of cellular stress response and a key trigger of inflammatory response during hepatic IRI. HRS treatment inhibited liver HMGB1 expression and release [48]. Besides, HRS also reduces the oxidative damage and inflammation of the liver caused by acute obstructive cholangitis by reducing the activity of MMP2/9 and inhibiting the hepatic gap junction and tight junction protein [49].

### Remodeling of glycolipid metabolism

The antioxidant and anti-inflammatory effects of molecular hydrogen have been well documented, but the free radical scavenging properties of molecular hydrogen cannot fully explain its extensive biological effects, especially in metabolic liver diseases such as hepatic steatosis, NASH, and MASLD. In people and animal models of metabolic liver diseases, it was observed that molecular hydrogen acted on the lipid profile to reduce cholesterol, triglycerides, and free fatty acids in circulation and liver tissue [15, 50]. 4% hydrogen inhalation significantly reduced high-fat diet (HFD)-induced circulation and hepatic accumulation of phospholipid oxidation products, with the most significant reduction in 1-palmitoyl-2-(9-oxo-nonanoyl) -s*n*-glycero-3-phosphatidylcholine (PONPC) [51]. The accumulation of lipids in the liver is thought to occur through a variety of mechanisms. Acetyl-CoA carboxylase (ACC) is a major component of de novo adipogenesis and catalyzes the production of malonyl-CoA. Sterol regulatory element binding transcription factor 1 (SREBP1) is a major regulator of FAS and other lipogenic proteins. Molecular hydrogen decreased the expression of SREBP-1c, ACC, and FAS, which inhibited the lipogenesis pathway and led to the reduction of liver fat accumulation[13]. L-Arabinose can effectively regulate the expression of fatty acid extension enzyme Elovl3 and fatty acid β-oxidation catalase Acadm in the liver of HFD mice [11]. Molecular hydrogen induced the Sirt1/AMPK pathway through the HO-1/AMPK/PPARα/PPARγ pathway to reverse fatty acid oxidation and lipogenesis in hepatocytes and inhibited palmitic acid-mediated abnormal fat metabolism [30]. Cluster of differentiation 36 (CD36) is a major fatty acid translocation enzyme that binds to long-chain fatty acids and promotes their transmembrane transport. Jackson [52] and Lio [28] observed that molecular hydrogen was associated with decreased expression of CD36 in hepatocytes, but Tsou's [53] experimental results were inconsistent with this, so whether CD36 was the target of molecular hydrogen was not conclusive. Lipid as a material basis, in which unsaturated fatty acids on the cell membrane will be attacked by ROS, resulting in lipid peroxidation. 4% hydrogen inhalation improves HDL protein composition and affects oxidative stress-related enzymes that bind to HDL, such as Lp-PLA2, PON-1, and LCAT, alleviating oxidative stress-induced lipid peroxidation [51]. NRF2 plays a vital role in each stage of its progression, including fat accumulation, inflammation, fibrosis, and hepatocellular carcinoma development. HRW can decrease the diameter of lipid droplets in HepG2 cells and reduce lipid peroxidation and free fatty acid (FFA)-induced oxidative stress by activating the AMPK/Nrf2/HO-1 pathway [53]. HRW improved lipid accumulation in hepatocytes exposed to free fatty acids via the miR-136/MEG3/Nrf2 axis [54]. In addition, Ma et al. [13] reported that molecular hydrogen also affects the expression of liver cholesterol synthesis genes SREBF2 and the downstream target gene 3-hydroxy-3-methylglutaryl-CoA reductase (HMGCR). The limited number of parameters evaluated has prevented definitive conclusions about molecular hydrogen's effects on cholesterol metabolic behavior and its possible mechanisms of action in metabolic liver diseases.

**Fig. 3:**
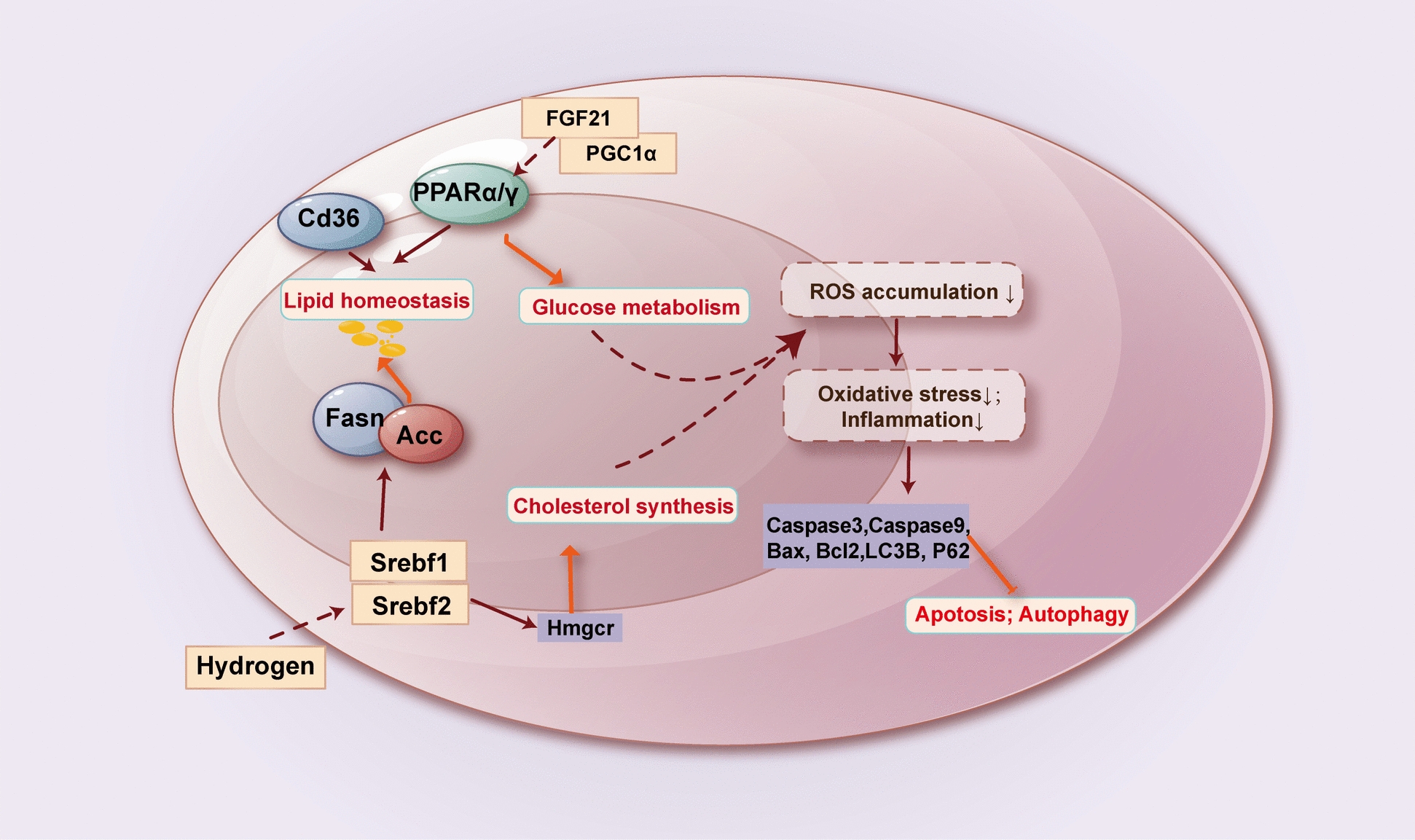
The potential regulatory mechanism of molecular hydrogen on liver glycolipid metabolism. ACC acetyl-CoA carboxylase; CD36 cluster of differentiation 36; FASN fatty acid synthase; FGF21 fibroblast growth factor 21; HMGCR 3-hydroxy-3-methylglutaryl-CoA reductase; PGC1α peroxisome proliferator-activated receptor gamma coactivator 1α; PPAR peroxisome proliferator-activated receptor; SREBF1/2 sterol regulatory element binding transcription factor 1/2

Altered regulation of hepatic glucose metabolism is a hallmark feature of steatosis liver diseases [55]. Molecular hydrogen has been shown to reduce fasting blood sugar and insulin levels and maintain glucose homeostasis in steatogenic liver diseases. HRW improved insulin sensitivity and increased energy metabolism by inducing liver fibroblast growth factor 21 (FGF21) to reduce hepatic lipid accumulation [56]. Kamimura [57] found that HRW regulates the 4HNE/Akt/Foxo1 signal transduction pathway, regulates the expression of Peroxisome Proliferator-activated receptor gamma coactivator 1α (PGC-1α), activates the PPARα pathway, promotes the transcription of FGF21, a metabolic gene related to fatty acid β-oxidation, and increases the consumption of fatty acids and glucose. HRS activated the expression of PPARα and PPARγ, inhibited the expression of fatty acid synthase, and improved liver steatosis and glucose sensitivity in rats induced by a high-fat and sugar diet [32] (Fig. 3). Although hydrogen has an overall protective effect on liver glycolipid metabolism, there is still a lack of in-depth research on the specific effects of hydrogen on key regulatory metabolic sites in liver tissue and its underlying mechanisms.

### Programmed cell death

The protective mechanism of molecular hydrogen against cell death in liver disease is not fully understood. Cell death is an important mechanism for maintaining morphogenesis and physiological homeostasis during development, including apoptosis, necrotic apoptosis, scorch death, iron death, and cell death associated with autophagy and unprogrammed necrosis. Molecular hydrogen decreased the proportion of TUNEL positive cells in liver injury lesions, decreased the expressions of hepatocyte apoptosis signals Caspase3/9 and Bax/Bcl2, and increased the expressions of KI67, PCNA, and Cyclin D1 to promote liver regeneration [38, 58, 59]. Tumor suppressor gene P53 can regulate apoptosis by promoting the expression of Bax and inhibiting the expression of Bcl-2. HRS exerted a protective role in promoting the recovery of liver function after liver IRI and hepatectomy in pigs by regulating the expression of P53, which inhibited hepatocyte apoptosis [60]. Moreover, inhalation of 2% H_2_ for 3 h could improve the expression of mitochondrial autophagy-associated proteins (P62, LC3B-II, and Tim23) and protect the liver of sepsis animals from mitochondrial damage [61]. Autophagy, including lipophagy, is also involved in regulating the degradation of lipid droplets. HRW could restore the expression level of LC3-II inhibited by FFA and decrease the expression of P62 to promote the autophagy activity and decomposition of lipid droplets in HepG2 cells [53]. The antioxidant and anti-inflammatory properties of molecular hydrogen contribute to cell protection, such as the activation of NF-κB, which is involved in the regulation of anti-apoptotic cascades and genes, including heme oxygenase, A20, and Bcl-2. Human and mouse caspase-1 are key activators of pyroptosis. Only Yan et al. mentioned that 2% hydrogen can reduce the expression of caspase-1 in liver [61]. Given that HMGB1 and IFN-γ have been proposed to be necessary for Caspase-1-dependent pyroptosis activation [62], it is reasonable to believe that molecular hydrogen has the potential to reduce pyroptosis-related liver inflammation.

### Intestinal barrier and gut microbiota (GM) profile

Alterations in the gut–liver axis, such as changes in gut microbiota and the levels of metabolites produced, are thought to be closely associated with MASLD. Intestinal barrier and GM are alternative targets for the anti-MASLD effect of molecular hydrogen. L-Arabinose [11] regulated the relative abundance of hydrogen-producing and hydrogen-consuming gut microbes and intestinal bacteria involved in metabolic syndrome in HFD mice, with Firmicutes, Bacteroidetes, and probiotic acidobacteria and bifidobacteria strains showing important roles. Xue et al. [63] proposed that inhalation of hydrogen can upregulate two tight junction proteins (ZO-1 and occlusion) on intestinal epithelial cells to improve the function of intestinal epithelial cells and change the relative abundance of Bacteroidetes and Firmicutes to improve the liver phenotype of rats fed HFD. Jin et al. [64] found that intestinal release of hydrogen supported by nanocapsules reversed HFD-induced reduction in Verrucomicrobia abundance at taxonomic phylum and Akkermansia abundance at taxonomic genus. These results highlight remodeling the gut microbiome as a potential mechanism for H_2_ treatment of liver metabolic dysfunction. However, the current evidence only observes changes in the structure and composition of GM, and there is a lack of fecal microbiota transplantation experiments, and additional in vitro studies of target cells may better reveal the mechanism.

## Conclusions

In this review, in addition to traditional hydrogen delivery methods such as hydrogen inhalation, drinking hydrogen-rich water, and injecting saturated hydrogen solutions, we focus on summarizing innovative hydrogen delivery technologies like hydrogen-rich diets and nano-hydrogen harvesting materials, as well as their advantages, and emphasize the necessity of interdisciplinary communication for the transformation of hydrogen delivery and storage. Second, it breaks through the previous limitation that H₂ mainly exerts its effects through anti-inflammation and maintaining mitochondrial function, and focuses on summarizing the latest mechanisms involved in metabolic remodeling, intestinal flora homeostasis, and cell protection in the application of hydrogen in liver diseases, aiming to provide more ideas and directions for the future application of hydrogen medicine in the field of liver diseases.

In basic experiments, molecular hydrogen has shown great potential in the treatment of liver inflammation and the progression of fatty metabolic liver disease. While initial results from clinical trials and studies are encouraging, further studies with larger sample sizes and rigorous methodology are needed to confirm these findings, particularly in viral hepatitis and liver cancer. The biological effects of hydrogen on liver disease cannot be fully explained by the existing biomedical theoretical system. The biological significance of molecular hydrogen in the remodeling of glucose and lipid metabolism homeostasis, cell protection, and intestinal flora regulation is not clear. Therefore, additional research is necessary to fully understand the exact mechanisms of molecular hydrogen to provide a beneficial and safe treatment for inflammatory as well as metabolic liver disease. It is strongly recommended that further in vitro and preclinical studies be encouraged in the future to identify the exact bioavailability, bioefficacy, and cell transduction signaling pathways of molecular hydrogen in chronic liver disease and its associated events. In the long run, hydrogen medicine should be based on the development idea of the integration of production, study, and research, and creatively integrate with other disciplines to achieve greater breakthroughs in health promotion.

## Supplementary Information

Additional file1 (XLSX 31 kb)

Additional file2 (XLSX 13 kb)

## Data Availability

No datasets were generated or analysed during the current study.

## Abbreviations

ACC: Acetyl-CoA carboxylase
AMPK: Adenosine 5′-monophosphate (AMP)-activated protein kinase
CAT: Catalase
CCH: Coral calcium hydride
CD36: Cluster of Differentiation 36
c-JNK: C-Jun N-amino-terminal kinase
FASN: Fatty acid synthase
FFA: Free fatty acid
FGF21: Fibroblast growth factor 21
GM: Gut microbiota
GPx: Glutathione peroxidase
HFD: High fat diet
HMGB1: High mobility group box 1 protein
HMGCR: 3-Hydroxy-3-methylglutaryl-CoA reductase
HO-1: Heme oxygenase 1
HRS: Hydrogen-rich saline
HRW: Hydrogen-rich water
IL: Interleukin
INFγ: Interferon-γ
iNOS: Inducible NOS
IRI: Ischemia–reperfusion injury.
MAPK: Mitogen-activated protein kinase
MASLD: Metabolic dysfunction-associated liver disease
MDA: Malondialdehyde
MMP2/9: Matrix metallopeptidase 2/9
NASH: Non-alcoholic steatohepatitis
NF-κB: Nuclear factor-κB
NRF2: Nuclear factor 2-related factor 2
NQO1: NAD(P)H quinone dehydrogenase 1
PdH: Pd hydroxide
PGC1α: Peroxisome proliferator-activated receptor gamma coactivator 1α
PONPC: 1-Palmitoyl-2-(9-oxo-nonanoyl) -sn-glycero-3-phosphatidylcholine
PPAR: Peroxisome proliferator-activated receptor
ROS: Reactive oxygen species
RNS: Reactive nitrogen species
SREBF1/2: Sterol regulatory element binding transcription factor 1/2
SOD: Superoxide dismutase
STAT3: Signal transducer and activator of transcription 3
TNFα: Tumor necrosis factor α
VEGF: Vascular endothelial growth factor
3-NT: 3-Nitrotyrosine
4-HNE: 4-Hydroxy-nonenal
8-OHdG: 8-Hydroxy-2-deoxyguanosine
